# Comparative clinical efficacy of photobiomodulation therapy and topical corticosteroids for oral lichen planus

**DOI:** 10.6026/973206300221522

**Published:** 2026-03-31

**Authors:** Rasakatla Hemanth, Madarapu Tejasvi, Eragattapu Sulochana, Pokkula Samskruthi, Javeria Sana, Raja Joanna, Pratik Surana

**Affiliations:** 1 Department of Oral Medicine and Radiology, Government Dental College & Hospital, Hyderabad, India; 2 Department of Pedodontics and Preventive Dentistry, Maitri College of Dentistry and Research Centre, Durg, Chhattisgarh, India

**Keywords:** Oral Lichen Planus (OLP), photobiomodulation therapy (PBMT), topical corticosteroids (TCS), randomized controlled trial, pain reduction, lesion severity, safety profile

## Abstract

Oral Lichen Planus (OLP) is a chronic inflammatory disorder impacting quality of life; this randomized, single-blind trial compared photobiomodulation therapy (PBMT) versus topical corticosteroids (TCS) for symptomatic OLP. Sixty patients received PBMT (660 nm, 4 J/cm^2^, thrice weekly) or 0.1% triamcinolone acetonide (thrice daily) for 4 weeks, with assessments at baseline, weeks 2, 4 and 8. At week 4, PBMT showed superior pain reduction (VAS: 4.2±0.8 vs. 3.1±0.9, p=0.002), greater lesion severity improvement (TSS reduction: 2.8±0.7 vs. 2.1±0.8, p=0.005) and higher clinical response (70% vs. 50%, p=0.031). PBMT maintained pain reduction advantage at week 8 (p=0.018) with no adverse events, while 10% of TCS patients reported mild irritation. Thus, PBMT demonstrates superior efficacy and safety over TCS for OLP management.

## Background:

Oral Lichen Planus (OLP) is a common, chronic, T-cell-mediated inflammatory disorder affecting the oral mucosa, with a prevalence estimated between 0.5% and 2.2% in the general population [1]. It presents clinically as reticular, papular, plaque-like, atrophic, erosive or ulcerative lesions, often characterized by bilateral symmetrical distribution, primarily on the buccal mucosa, tongue and gingiva [2]. Symptoms range from asymptomatic to significant pain, burning sensation, dysphagia and dysgeusia, severely impacting oral function, nutritional intake and overall quality of life [3]. While the etiology remains incompletely understood, it is widely accepted as an immune-mediated process involving autoreactive cytotoxic T-cells targeting basal keratinocytes, triggered by factors such as stress, genetic predisposition, infections or certain medications [4]. The primary goals of OLP management are to alleviate symptoms, promote healing of erosive/ulcerative lesions, reduce inflammation and prevent malignant transformation, although the latter remains controversial and requires long-term monitoring [5]. Topical corticosteroids (TCS), such as triamcinolone acetonide, fluticasone propionate and clobetasol propionate, are universally recommended as first-line therapy due to their potent anti-inflammatory and immunomodulatory effects [6]. However, their long-term use is associated with potential local adverse effects including mucosal atrophy, secondary candidiasis and taste disturbances, raising concerns about patient compliance and safety [7]. Furthermore, a significant proportion of patients exhibit suboptimal response or relapse upon discontinuation [8]. In recent years, there has been growing interest in non-pharmacological therapeutic modalities. Photobiomodulation therapy (PBMT), previously known as low-level laser therapy (LLLT), utilizes non-thermal light in the red or near-infrared spectrum to modulate cellular processes [9]. PBMT has demonstrated biostimulatory effects, including enhanced tissue repair, reduced inflammation, analgesia and modulation of immune responses, primarily through mitochondrial cytochrome c oxidase activation and subsequent downstream signaling cascades [10]. Several studies have reported promising results with PBMT in managing OLP, showing reductions in pain scores and clinical lesion severity with minimal to no side effects [11, 12]. However, the majority of existing literature consists of case series, small cohort studies, or comparisons with placebo. While PBMT is increasingly used in clinical practice, robust evidence directly comparing its efficacy and safety to the current gold standard TCS in a well-designed randomized controlled trial (RCT) remains limited [13]. This comparative evidence gap hinders definitive clinical recommendations regarding the optimal first-line approach. Therefore, it is of interest to conduct a randomized, single-blind, controlled clinical trial to directly compare the clinical efficacy and safety profile of PBMT versus a standard topical corticosteroid (0.1% triamcinolone acetonide) in the management of symptomatic OLP.

## Materials and Methods:

Based on a pilot study and previous literature, the primary outcome was the change in Visual Analog Scale (VAS) pain score from baseline to week 4. Assuming a clinically significant difference of 1.5 units in mean VAS reduction between groups, a standard deviation of 1.8, 80% power and a two-sided alpha level of 0.05, a minimum sample size of 26 participants per group was calculated. Accounting for a potential 15% dropout rate, the target sample size was set at 30 participants per group (total N=60).

## Participants:

Consecutive patients presenting to the Oral Medicine Clinic of a tertiary care hospital between January 2023 and December 2023 were screened for eligibility.

[1] Inclusion Criteria: (1) Adults aged 18-75 years; (2) Clinically diagnosed with symptomatic OLP (reticular, erosive, or atrophic forms) confirmed by histopathological examination according to WHO diagnostic criteria; (3) Presence of at least one symptomatic lesion with a baseline VAS pain score ≥ 4 cm; (4) Willingness to comply with the study protocol and attend follow-up visits.

[2] Exclusion Criteria: (1) Presence of other significant oral mucosal diseases (e.g., pemphigus, pemphigoid, leukoplakia, oral candidiasis); (2) Use of systemic corticosteroids, immunosuppressants or other topical treatments for OLP within the preceding 4 weeks; (3) History of malignant transformation in oral lesions; (4) Pregnancy or lactation; (5) Known photosensitivity disorders or use of photosensitizing medications; (6) Severe systemic uncontrolled diseases (e.g., uncontrolled diabetes, autoimmune disorders); (7) History of head and neck radiotherapy.

##  Randomization and blinding:

Eligible participants were randomly assigned to either the PBMT group or the TCS group using a computer-generated block randomization sequence (block size of 4) prepared by a statistician not involved in patient recruitment or assessment. Allocation concealment was maintained using sealed, opaque, sequentially numbered envelopes opened by the treating clinician after baseline assessment. Due to the nature of the interventions, participants could not be blinded to group assignment. However, the outcome assessor (a calibrated oral medicine specialist) and the data analyst were blinded to the group allocation.

## Interventions:

[1] PBMT Group: Participants received PBMT using a class 3B diode laser device (MedLase® Pro, Wavelength: 660 nm ± 10 nm, Continuous Wave mode, Output Power: 100 mW). The laser probe was held in contact with the lesion surface, perpendicular to the tissue, without pressure. The energy density delivered per point was 4 J/cm^2^. Treatment was applied to all symptomatic lesions, covering the entire lesion area with point's spaced approximately 1 cm apart. Each session lasted approximately 5-10 minutes depending onlesion size. PBMT was administered three times per week (e.g., Monday, Wednesday, Friday) for four consecutive weeks (total 12 sessions).

[2] TCS Group: Participants received 0.1% triamcinolone acetonide oral paste (Kenalog® in Orabase). Participants were instructed to apply a thin layer (approximately 0.5 cm ribbon) directly onto the symptomatic lesions using a clean finger or cotton swab, three times daily (after meals and before bedtime), for four consecutive weeks. They were advised not to eat or drink for at least 30 minutes after application.

## Outcome measures:

Assessments were performed at baseline (T0), week 2 (T1), week 4 (end of treatment, T2) and week 8 (follow-up, T3) by the blinded assessor.

[1] Primary outcome:Pain Intensity: Measured using a 10-cm Visual Analog Scale (VAS), where 0 represented "no pain" and 10 represented "worst imaginable pain". Participants marked their current pain level.

[2] Secondary outcomes:1) Clinical Lesion Severity: Assessed using the Thongprasom Sign Score (TSS), a validated 5-point scale: 0 = no lesion, normal mucosa; 1 = mild white striae only; 2 = white striae with erythematous area <1 cm^2^; 3 = white striae with erythematous area >1 cm^2^; 4 = white striae with erosive/ulcerative area <1 cm^2^; 5 = white striae with erosive/ulcerative area >1 cm^2^.2) Clinical Response Rate: Defined as the proportion of participants achieving ≥50% reduction in TSS score from baseline to week 4 (T2).3) Signs of inflammation: Presence and extent of erythema and ulceration were recorded qualitatively (absent, mild, moderate, severe) and quantitatively by measuring the surface area (mm^2^) of the largest erosive/ ulcerative lesion using a periodontal probe. 4) Adverse events: Participants were questioned and examined for any local or systemic adverse events at each visit. Specific attention was paid to mucosal irritation, burning sensation, taste alteration and signs of candidiasis in the TCS group and any unusual sensations or tissue reactions in the PBMT group.

## Statistical analysis:

Data were analyzed using SPSS software version 28.0 (IBM Corp., Armonk, NY, USA).

## Results:

Of the 82 patients screened, 60 met the eligibility criteria and were randomized equally into the photobiomodulation therapy (PBMT) and topical corticosteroid (TCS) groups (n = 30 each). All participants completed the 4-week treatment phase, while one participant in the TCS group was lost to follow-up at week 8. Baseline demographic and clinical characteristics were comparable between groups, with no significant differences in age, sex distribution, clinical form of oral lichen planus, or baseline pain and lesion severity scores (Table 1 - see PDF). Both groups showed significant reductions in pain intensity (VAS) and lesion severity (TSS) from baseline at all-time points (p < 0.001). However, the PBMT group demonstrated significantly greater pain reduction than the TCS group at weeks 2, 4 and 8 (p < 0.05). The mean reduction in VAS score from baseline to week 4 was significantly higher in the PBMT group compared with the TCS group (p = 0.002) (Table 2 - see PDF). Clinical lesion severity improved significantly in both groups over time. The PBMT group showed significantly lower TSS scores at weeks 2 and 4 compared with the TCS group (p < 0.05), while the difference at week 8 was not statistically significant. A higher proportion of participants in the PBMT group achieved a ≥50% reduction in TSS at week 4 compared with the TCS group (70.0% vs. 50.0%; p = 0.031). Both treatment modalities resulted in significant reductions in erosive lesion size; however, the reduction was significantly greater in the PBMT group at week 4 (p = 0.008). No adverse events were reported in the PBMT group. In the TCS group, three participants experienced mild local irritation, with no cases of candidiasis or mucosal atrophy observed.

## Discussion:

This randomized controlled trial provides robust evidence demonstrating the superior efficacy of PBMT compared to a standard topical corticosteroid (0.1% triamcinolone acetonide) in alleviating pain and improving clinical signs in patients with symptomatic OLP over a 4-week treatment period. The significant advantages observed in pain reduction, lesion severity improvement and clinical response rate, coupled with the absence of adverse events in the PBMT group, highlight its potential as a first-line therapeutic option. The findings align with the proposed mechanisms of action for PBMT. The 660 nm wavelength effectively penetrates oral mucosa, stimulating mitochondrial cytochrome c oxidase, leading to increased ATP production, modulation of reactive oxygen species and activation of transcription factors [14]. This cascade results in reduced pro-inflammatory cytokine production (e.g., TNF-α, IL-1β, IL-6), decreased neutrophil infiltration and promotion of anti-inflammatory mediators [15]. Furthermore, PBMT enhances microcirculation and accelerates tissue repair processes, contributing to the faster healing of erosive and ulcerative lesions observed in this study [16]. The analgesic effect is attributed to reduced inflammation, decreased nerve conduction velocity and increased endorphin release [17]. These multifaceted actions likely explain the more rapid and pronounced symptomatic and clinical improvements seen with PBMT compared to TCS, which primarily acts through glucocorticoid receptor-mediated suppression of inflammatory gene expression [18]. The superior pain reduction achieved with PBMT is particularly significant, as pain is the primary driver for seeking treatment and the major factor impacting quality of life in OLP patients [19].

The significant reduction in pain observed in the present study is in agreement with the findings of Mahuli *et al.* who reported superior pain relief with photobiomodulation therapy compared to topical corticosteroids in patients with oral lichen planus [20]. The significantly higher clinical response rate (≥50% TSS reduction) and greater reduction in TSS scores and erosive lesion size in the PBMT group further underscore its efficacy in resolving the underlying inflammatory pathology. While TCS also produced significant improvements, consistent with its established role as first-line therapy [6], the magnitude of benefit was consistently lower than that achieved with PBMT. This suggests that PBMT may offer a more potent anti-inflammatory effect in the context of OLP. The comparable TSS scores at the 8-week follow-up, despite the initial advantage of PBMT, indicate that both interventions provide sustained benefit, but PBMT achieves a superior outcome during the active treatment phase. The excellent safety profile of PBMT is a major advantage. The absence of any reported adverse events contrasts with the 10% incidence of mild local irritation in the TCS group. While more serious side effects like candidiasis or atrophy were not observed in this relatively short-term study, they remain well-documented risks associated with long-term TCS use [7]. PBMT avoids these risks entirely, making it particularly suitable for patients requiring prolonged therapy, those prone to candidiasis, or individuals concerned about corticosteroid side effects. This safety aspect is crucial for chronic conditions like OLP where long-term management is often necessary. The results of this study contribute significantly to the existing literature. Previous investigations have largely supported the efficacy of PBMT in OLP management, often comparing it to placebo or other alternative therapies [11, 12 and 21]. However, direct comparisons with the current gold standard TCS have been scarce and often methodologically limited [13]. A recent study conducted by Mohamed *et al.* highlighted the need for more high-quality RCTs comparing PBMT directly with TCS [22]. This trial addresses that gap effectively. The findings are consistent with a smaller RCT by De Souza *et al.* (2010) which also reported greater pain reduction with PBMT compared to clobetasol propionate, although their sample size was smaller and follow-up shorter [23]. The strengths of this study include its randomized, controlled, single-blind design, adequate sample size based on power calculation, use of validated outcome measures (VAS, TSS), standardized protocols for both interventions and intention-to-treat analysis. The inclusion of a follow-up period (week 8) provides valuable data on the durability of the treatment effects. However, certain limitations should be acknowledged. The single-blind design (participant unblinded) is inherent to the nature of the interventions but could introduce bias in subjective reporting, although the blinded assessment of outcomes mitigates this concern. The study duration was relatively short (4 weeks treatment + 4 weeks follow-up); longer-term studies are needed to assess the sustainability of PBMT effects and its potential to prevent relapse over extended periods. The study focused on symptomatic OLP; results may not be generalizable to asymptomatic reticular forms. While histopathological confirmation was used, molecular markers of inflammation were not assessed, which could provide deeper mechanistic insights. Finally, the study utilized a specific PBMT protocol (660 nm, 4 J/cm^2^); different wavelengths, doses or application frequencies might yield varying results, warranting further research on optimal parameters. It can be summarized that the therapeutic benefits of photobiomodulation in oral lichen planus are likely mediated through its immunomodulatory and anti-inflammatory actions, promotion of tissue regeneration, and effective analgesic effect, leading to significant reduction in pain and clinical symptoms [24, 25- 26]. Future research should prioritize long-term RCTs comparing PBMT with various TCS formulations and potencies, as well as other second-line agents like calcineurin inhibitors. Studies investigating combination therapies (e.g., PBMT + low-dose TCS) could explore synergistic effects and potential for corticosteroid-sparing regimens. Research into standardized PBMT protocols and cost-effectiveness analyses are also needed to guide clinical implementation.

## Conclusion:

We show that photobiomodulation therapy (PBMT) using a 660 nm diode laser is significantly more effective than 0.1% triamcinolone acetonide oral paste in reducing pain and improving clinical signs of oral lichen planus over a 4-week treatment period. PBMT achieved faster and greater symptomatic relief, a higher clinical response rate and superior resolution of erosive lesions compared to the standard topical corticosteroid.

## Advancement to knowledge:

his study advances current knowledge by comparing the effectiveness of photobiomodulation therapy with topical corticosteroids in oral lichen planus management, highlighting the potential of photobiomodulation as a safe and effective alternative.
